# The application of rituximab during the conditioning regimen prevents Epstein - Barr virus infection following rATG-based haploidentical hematopoietic stem cell transplantation in the era of letermovir for cytomegalovirus prophylaxis

**DOI:** 10.3389/fimmu.2026.1795239

**Published:** 2026-06-19

**Authors:** Jinxia Hao, Tongxin Zhang, Juan Ren, Xiaoning Wang

**Affiliations:** 1 Department of Internal Medicine, Xi’an Jiaotong University Hospital, Xi’an, Shaanxi, China; 2 Department of Hematology, The First Affiliated Hospital of Xi’an Jiaotong University, Xi’an, Shaanxi, China

**Keywords:** acute graft-versus-host disease, EBV viremia, haploidentical hematopoietic stem cell transplantation, post-transplant lymphoproliferative disorders, rituximab

## Abstract

**Background:**

In the era of letermovir for cytomegalovirus (CMV) prophylaxis, several centers reported that the incidence of Epstein-Barr virus (EBV) infection were significantly increased.

**Objective:**

To investigate the efficacy and safety of rituximab administration during conditioning regimen following haploidentical hematopoietic stem cell transplantation(haplo-HSCT)in the prevention of post-transplant EBV infection.

**Methods:**

We conducted a retrospective analysis of 100 patients with acute leukemia or myelodysplastic syndrome who underwent haplo-HSCT. Patients in observation group(R group) received rituximab (375 mg/m²) on day -3 before transplantation due to the presence of donor-specific antibody (MFI ≥ 2000) (n = 25) and patients in control group (C group) did not receive rituximab (n = 75) and donor-specific antibody was low (MFI < 2000). The primary objectives were the incidence of EBV-DNA viremia and PTLD within one-year post-transplantation. Secondary objectives included the incidence of CMV infection, cumulative incidence of acute graft-versus-host disease (aGVHD) and chronic GVHD, 100-day non-relapse mortality (NRM), progression-free survival (PFS), and overall survival (OS).

**Results:**

No significant differences were observed in baseline characteristics between the two groups except for primary disease. When compared with the C group, patients in R group exhibited a lower cumulative incidence of EBV viremia within one-year post - transplantation (4.00% vs. 22.67%, P = 0.049) and a lower incidence of aGVHD (28% vs. 50.67%, P = 0.048). There was a trend toward reduction of PTLD in the R group compared with C group (0% vs. 10.67%, P = 0.089).There were no significant differences of the incidence of CMV viremia (24% vs. 13.33%, P = 0.208), cGVHD (16% vs. 12%, P = 0.607), and 100 - day NRM (4.0% vs. 10.67%, P = 0.313) between two groups. The 2-year OS rates in the R group and C group were 83.8% ± 0.086% and 81.9% ± 0.050% respectively (P = 0.360). The 2-year PFS rates in the R group and C group were 83.8% ± 0.086% and 72.6% ± 0.068% respectively (P = 0.360).

**Conclusion:**

The combined use of rituximab during the conditioning regimen may be regarded as an effective strategy for preventing EBV reactivation after rATG - based haplo - HSCT in the era of letermovir for CMV prophylaxis.Prospective randomized controlled trials are still required to further validate the reliability of the results.

## Introduction

Haploidentical hematopoietic stem cell transplantation (haplo-HSCT) is a common curative strategy for patients with hematological malignancies. Epstein-Barr virus (EBV) reactivation is a common complication post-transplantation, especially in the era of letermovir prophylaxis for cytomegalovirus(CMV) reactivation (1–3). There is an urgent need to identify effective prevention and treatment methods. The traditional “preemptive treatment” strategy for EBV viremia involves the use of CD20 monoclonal antibodies upon detection of high EBV loads to prevent post-transplant lymphoproliferative disorders (PTLD) (4). In recent years, studies have explored integrating this approach into conditioning regimens for “preventive” application, aiming to eliminate B lymphocytes early and reduce the EBV viral reservoir (5). However, the exact preventive efficacy of this strategy against EBV reactivation, as well as its potential impact on immune reconstitution, graft versus host disease (GVHD), and long-term survival in the context of haplo-HSCT with ATG-based GVHD prophylaxis, still requires further evidence.

This study aims to evaluate the impact of prophylactic application of rituximab in conditioning regimen for haplo-HSCT on the incidence of EBV infection within one-year post-transplantation, and comprehensively analyze its effects on GVHD and patient survival outcomes, thereby providing evidence for optimizing clinical prophylactic strategies.

## Materials and methods

### Patients

A retrospective analysis was conducted on patients who underwent haplo-HSCT at the Department of Hematology, the First Affiliated Hospital of Xi’an Jiaotong University, from January 2024 to September 2025. Inclusion criteria were as follows:Age ≥18 years, received haplo-HSCT for first time, use myeloablative conditioning regimen containing rabbit anti-thymocyte globulin (rATG) for GVHD prophylaxis, complete EBV DNA monitoring data. Exclusion criteria were included active EBV infection prior to transplantation, concurrent other uncontrolled active infections, incomplete clinical data.Enrolled patients were divided into two groups.Patients received rituximab 375mg/m^2^ during conditioning were defined as (R Group). The control group (C Group) were screened from patients who did not receive rituximab at the same time, with a 1:3 ratio. Matching factors included: age (± 5 years), disease status at transplantation (complete remission vs. no response), and donor relationship.All patients provided the written consents and the study was approved by the Ethics Committee of the First Affiliated Hospital of Xi’an Jiaotong University.

### Conditioning regimen

Modified BuCy2 protocol, consisting of cytarabine 4g/m² on days -9 to -8, busulfan 0.8mg/kg every 6 hours on days -7 to -5, cyclophosphamide 1.8g/m² on days -4 to -3, semustine 250mg/m² on day -3. TBF protocol, consisting of thiotepa 5mg/kg d-7 to-6, busulfan 0.8mg/kg every 6 hours on days -5 to -3, fludarabine 30mg/m² on days -5 to -2.TBI or TMI based conditioning regimen, consisting of 8-12Gy total body irradiation or total marrow irradiation.Patients in R group received rituximab (375 mg/m²) on day -3 before transplantation due to the presence of donor-specific antibody (MFI ≥ 2000).

### Graft versus host disease prophylaxis and treatment

A combination of mycophenolate mofetil (MMF), cyclosporine (CSA), and short-term methotrexate (MTX) was employed for GVHD prophylaxis in all patients and antithymocyte globulin (rATG) 2.5mg/kg/day on days -5 to -2 for GVHD prophylaxis.

Acute GVHD (aGVHD) and chronic GVHD (cGVHD) were diagnosed according to standard references (6, 7). For patients experiencing aGVHD, immediate first-line treatment involved administering methylprednisolone at a dose of 1–2 mg/kg.d.In cases where methylprednisolone was ineffective or dependency occurred, second-line therapies such as ruxolitinib, anti-CD25 monoclonal antibodies, among others, were administered. The primary treatment for cGVHD involved the use of methylprednisolone and/or CSA as the first-line approach.

### EBV and CMV monitoring, definitions, prophylaxis and antiviral therapy

Prophylaxis for CMV infection with ganciclovir was administered from beginning of conditioning to the day before transplantation, and acyclovir prophylaxis was given from the day of neutrophil recovery to one-year post-transplantation.Letermovir was administrated after the infusion day of transplantation to 100days post-transplantation.

All patients underwent peripheral plasma CMV-DNA or EBV-DNA load testing via real-time quantitative PCR twice per week post-transplantation until day 90. From day +90 onward, monitoring was conducted every 1–2 weeks until day +180 and every one month until one-year post-transplantation.

According to our internal standards, CMV or EBV viremia is defined as two consecutive CMV-DNA or EBV-DNA tests showing levels exceeding 500 copies/ml, or a single CMV-DNA or EBV-DNA test result exceeding 1, 000 copies/ml.

In cases of EBV viremia or PTLD, immunosuppressant tapering and rituximab therapy were initiated.For CMV viremia, ganciclovir or foscarnet sodium was given. PTLD is definitively diagnosed through lymph node biopsy pathology.

### Outcomes and definitions

Primary endpoint was cumulative incidence of EBV viremia or PTLD within one-year post-transplantation.

Secondary endpoints were hematopoietic recovery, the cumulative incidence of aGVHD (graded according to the Glucksberg criteria), cumulative incidence of cGVHD (graded according to NIH criteria), overall survival (OS), non-relapse mortality (NRM), and progression free survival(PFS).

Neutrophil engraftment was defined as a consecutive 3-day absolute neutrophil count (ANC) > 0.5 × 10^9^/L, while platelet engraftment was defined as a consecutive 7-day platelet count (PLT) > 20 × 10^9^/L without requiring platelet transfusions. OS was defined as the period from transplantation to death from any cause or last follow-up.PFS was defined as the period from transplantation to relapse, death, or last follow-up.NRM was death due to transplant-related causes rather than disease recurrence.

### Statistical analysis

Statistical analysis was performed using R4.4.3 software. Measurement data were expressed as median (range) and analyzed by the Mann-Whitney U test; count data were presented as rates (%) and analyzed by the χ² test or Fisher’s exact test. Survival analysis was conducted using the Kaplan-Meier method, with the Log-rank test employed to compare intergroup differences. A P-value <0.05 was considered statistically significant.

## Results

### Patient clinical characteristics

A total of 100 patients were enrolled, including 25 in R Group and 75 in C Group. The two groups were well-matched in baseline characteristics such as age, gender, disease status, donor-recipient relationship, HLA mismatch number, conditioning regimens (excluding rituximab), and GVHD prophylaxis (P>0.05), except for the primary disease(**Table 1**).

**Table 1:** Baseline characteristics of patients in two groups.

Patients	R group	C group	P value
Sex(Male/Female)	12/13	41/34	0.563
Median age(years, range)	41 (15-63)	37 (16-66)	0.482
Disease			0.005*
AML	19	31	
ALL	3	36	
MDS	3	8	
Status before transplantation			0.308
MRD-CR	17	44	
MRD+CR	3	20	
PR/NR	5	11	
Relationship of donor-recipient			0.353
Sibling	6	27	
parents	5	14	
children	14	30	
others	0	4	
Blood type of donor-recipient			0.373
compatibility	9	20	
incompatibility	16	55	
Conditioning regimen			0.067
TBF based	7	9	
Bucy based	16	64	
TMI/TBI based	2	2	
Mononuclear infused	7.4 (4.65-11.88)	6.58 (4.25-12.02)	0.254
CD34+ cells infused	8.79 (3.45-17.99)	6.9 (2.015-14.17)	0.167

R, rituximab; C, control; AML, acute myeloid leukemia; ALL, acute lymphoblastic leukemia; MDS, myelodysplastic syndrome; MRD, minimal Residual Disease; CR, complete remission; TMI, total marrow irradiation; TBI, total body irradiation.

*There was significant difference of primary disease between two groups.

### Hematopoietic recovery

The median time for neutrophil engraftment in R and C group were 11 days (range: 9–15 days) and 10 days(range:8–16 days). For platelet engraftment, the median time for R group was 11 days (range: 9–15) and two patients in C group had platelet engraftment delay and the median time for platelet engraftment of others in C group was12 days (range: 8–29).There was no significant difference of neutrophil engraftment and platelet engraftment between two groups(P=0.134, 0.271).

### Overview of EBV infection, treatment, and outcome

**Figure 1:**
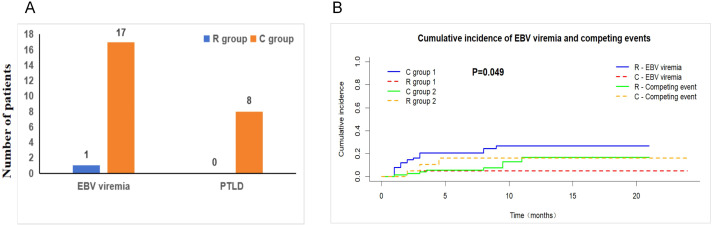
EBV viremia and PTLD in two groups. **(A)** Number of patients with EBV and PTLD in two groups; **(B)** Cumulative incidence of EBV viremia in two groups.

Among all patients, 18 cases experienced EBV viremia, with a median onset of EBV viremia at 1.5 months (range: 1–8 months) post-transplantation. The median viral load of EBV viremia was 3.32 × 10^3^ copies(range 1× 10^3^ to 6.0 × 10^5^copies). Eight patients had PTLD withe a median onset at 49 days post- transplantation(range:49–90 days).In Group R, one patient had concurrent EBV viremia, and no PTLD occurred. In Group C, 17 patients had EBV viremia, and 8 developed PTLD(**Figure 1A**).Compared with the C group, patients in R group had a significantly lower cumulative incidence of EBV viremia within one-year post-transplantation (4.00% vs.22.67%, P = 0.049)(**Figure 1B**) and a trend toward reduction of PTLD (0% vs. 10.67%, P = 0.089).Due to differences in the proportion of disease types in the baseline data, analysis was conducted in the AML/MDS subgroup. No cases of EBV viremia were observed with AML/MDS subgroup in the R group, while 8 cases were observed with AML/MDS subgroup in the C group (0/22 vs. 8/39; P = 0.023). All patients with EBV viremia were treated with rituximab.One was died of hemophagocytic syndrome due to EBV infection.The quantitative EBV-DNA levels of other patients all turned negative after treatment.No cases of PTLD were observed with AML/MDS subgroup in the R group, while 4 cases were observed with AML/MDS subgroup in the C group (0/22 vs. 4/39; P = 0.120). No cases of PTLD were observed with ALL subgroup in the R group, while 4 cases were observed with ALL subgroup in the C group (0/3 vs. 4/36; P = 0.542).

### Overview of other virus infection, treatment, and outcome

Before transplantation, both donor and recipient CMV-DNA quantification levels were below the detection range (<500 copies/ml). By the end of the follow-up period, CMV infection occurred in 6 out of the 25 patients (24%) in R group and 10 out of the 75 patients (13.33%) in C group, and there was no significant difference of CMV viremia between two groups(P = 0.208). All 6 patients with CMV viremia in Group R became negative after treatment with ganciclovir. Among the 10 patients in Group C, 1 developed ganciclovir resistance but turned negative after treatment with maribavir. The remaining patients all became negative after treatment with ganciclovir.

One patient in R group and 11patients in C group had hemorrhagic cystitis with BKV infection and There was no significant difference of BKV infection between two groups(P=0.155).

### Graft versus host disease

Seven patients in R group experienced aGVHD(2 cases with grade I aGVHD, 3 cases with grade II aGVHD, one with grade III aGVHD and one with grade IV aGVHD)and four had chronic GVHD.Thirty-eight patients in C group experienced aGVHD(10 cases with grade I aGVHD, 17 cases with grade II aGVHD, 6 cases with grade III aGVHD and 5 cases with grade IV aGVHD)and nine had chronic GVHD.When compared with the C group, patients in the R group exhibited a significantly lower cumulative incidence of grade I - IV aGVHD (28% vs. 50.67%, P = 0.048) and there was no significant difference of cGVHD between two groups(16% vs. 12%, P = 0.607).

### Survival

**Figure 2:**
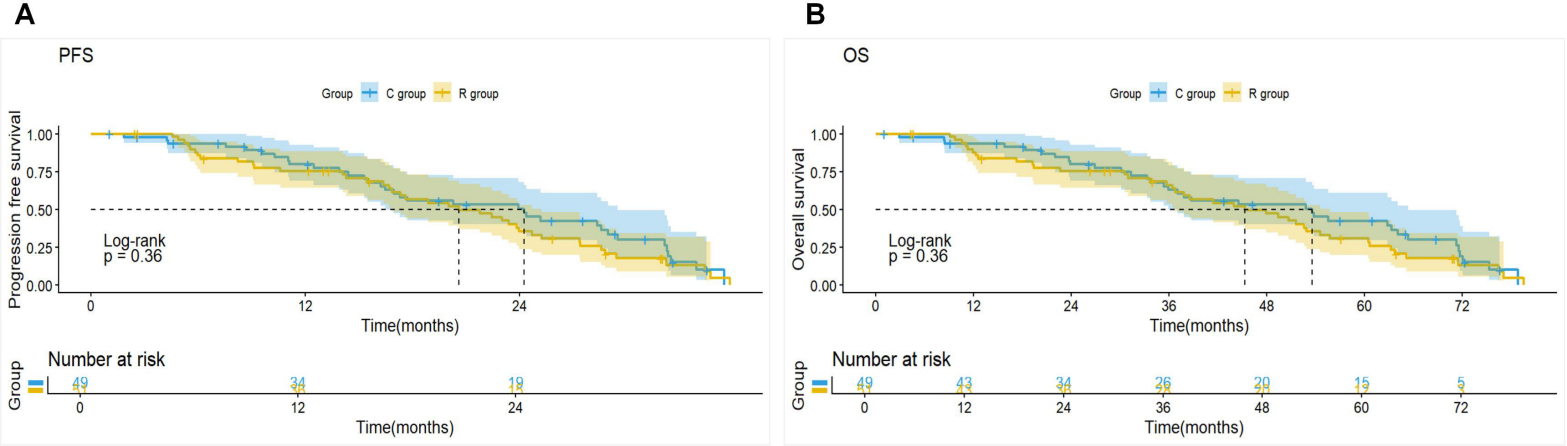
Progression free survival and overall survival of patients in two groups. **(A)** PFS; **(B)** OS.

All patients were followed up until Janurary 1^st^, 2026.The median follow-up period was 9 months(1-24months), with no patients lost to follow-up.The 2-year OS in the R group was 83.8% ± 0.086%, compared to 81.9% ± 0.050% in the C group (P = 0.360). The 2-year PFS in the R was 83.8% ± 0.086%, compared to 72.6% ± 0.068% in the C group (P = 0.360)(**Figure 2**).There was no difference of within100-day NRM between two groups (4.0% vs. 10.67%, P = 0.313).

## Discussion

With the widespread application of letermovir (LTV) for CMV prophylaxis following haplo-HSCT, the rate of CMV reactivation has significantly decreased. However, this has also introduced new clinical challenges—the potential corresponding increase in the risk of reactivation of other herpesviruses, particularly Epstein-Barr virus (EBV).A propensity score matching study on haploidentical HSCT demonstrated that patients receiving LTV prophylaxis had a significantly higher cumulative incidence of EBV viremia within 100 days post-transplantation compared to those without LTV (38.7% vs.13.7%, P <0.001), with LTV prophylaxis being an independent risk factor for EBV viremia (HR = 2.69). Additionally, the 1-year cumulative incidence of relapse post-HSCT was notably higher in the LTV group than in the non-LTV group (13.2% vs. 6.1%, P = 0.032). In multivariate analysis, LTV prophylaxis was an independent risk factor for relapse (HR = 2.56, P = 0.024).The study also revealed that Lymphocyte subset counts and functions post-transplantation were significantly lower in the LTV group than in the non-LTV group which suggested that LTV may delay immune reconstitution, particularly the recovery of virus-specific T cells (2). Xin Kong et al. also reported that increased incidence of EBV reactivation may be associated with LTV prophylaxis for CMV after haploidentical HSCT (8).Jingtao Huang et al. reported letermovir prophylaxis did not increase the risk of EBV DNAemia in allo-HCT recipients, it was associated with a higher incidence of PTLD (9). These data indicate that in an era of widespread LTV use, the risk of EBV reactivation is significantly increased, potentially accompanied by poorer immune reconstitution and a higher risk of recurrence. Therefore, for high-risk patients receiving LTV prophylaxis, enhanced EBV monitoring and targeted prophylactic measures should be considered.

Currently, the prevention of EBV reactivation primarily focuses on three aspects: antiviral therapy, immunomodulation, and preemptive treatment. Antiviral agents (e.g., ganciclovir, valganciclovir) have limited efficacy in preventing EBV infection, and long-term use may lead to adverse effects such as bone marrow suppression. Rational dose reduction of immunosuppressants is fundamental, but it must balance the risk of GVHD. Therefore, preemptive treatment has become the cornerstone of current clinical practice. According to the EBV-PTLD prevention and preemptive treatment recommendations, high-risk patients should initiate preemptive treatment upon the appearance of two consecutive peripheral blood EBV-DNA positive tests. Rituximab is considered the most critical preemptive treatment option, and its combination with immunosuppressant dose reduction is recommended. Centers with adequate resources may also consider EBV-specific cytotoxic T lymphocyte (EBV-CTL) infusion (10). Additionally, rituximab preemptive treatment guided by molecular surveillance has been proven to effectively reduce the incidence of EBV-lymphoproliferative disease and associated mortality (5).

Based on the clearance of CD20+ B cells and reduction of the EBV latent pool, rituximab may be used for prophylaxis of EBV infection in high risk of EBV infection patients.Patel C^[12]^ reported that 43 allo-HSCT recipients undergoing T-cell depletion with alemtuzumab received 1 dose of rituximab within 6 months before HLA-identical allo-HSCT and 43 patients did not receive pre-HSCT rituximab.EBV reactivation at day 180 occurred in 23 (53%) patients without prior rituximab exposure versus 0 patients with pre-HSCT rituximab exposure (P <.0001). Similarly, 6 patients without prior rituximab exposure developed PTLD at 1 year compared to no cases of PTLD among patients receiving pre-HSCT rituximab.In our study, it also showed that compared with the control group, patients in the rituximab group exhibited a significantly lower cumulative incidence of EBV viremia within one-year post - transplantation (4.00% vs. 22.67%, P = 0.049) and a trend toward reduction of PTLD (0% vs. 10.67%, P = 0.089).It suggested that pre-HSCT rituximab for EBV prophylaxis may be effective.

Certainly, the application of rituximab also requires weighing its potential risks, such as infections, hypogammaglobulinemia and impacts on the complications of HSCT. Patel C^[12]^ reported that there was no difference in neutrophil engraftment, incidence of infections, or aGVHD at day 180 between patients with or without pre-HSCT rituximab exposure. There was a delay in time to platelet engraftment in the rituximab cohort (median 16 [IQR 15-20] days versus 15 [IQR 14-17] days; P = .04). In our study, we found that compared with the C group, patients in the R group exhibited a significantly lower incidence of grade I - IV aGVHD (28% vs. 50.67%, P = 0.048). There were no significant differences in the incidence of CMV viremia (24% vs. 13.33%, P = 0.208), cGVHD (16% vs. 12%, P = 0.607), and 100 - day non - relapse mortality (NRM) (4.0% vs. 10.67%, P = 0.313). The 2-year OS rates in the R group and C group were 83.8% ± 0.086% and 81.9% ± 0.050% respectively (P = 0.662). The 2-year PFS rates in the R group and C group were 83.8% ± 0.086% and 72.6% ± 0.068% respectively (P = 0.876). It suggested that administration of pre-HSCT rituximab before allo-HSCT in patients receiving HSCT may not increase the incidence of infection. The varying outcomes of aGVHD may be partially attributable to differences in transplant types indifferent study.

The study also had certain limitations. Firstly, it was a retrospective study, and the patients in the R group with positive DSA and the primary diseases of the two groups were not comparable. Secondly, the small sample size of the study might affect the conclusion. Thirdly, further prospective trials are still required to verify the timing and dosage of rituximab for the prophylaxis of EBV infection.

## Conclusion

The addition of rituximab to the conditioning regimen for haplo - HSCT could effectively reduced the risk of early post - transplant EBV reactivation and decreased the incidence of aGVHD, without exerting a significant influence on CMV reactivation, cGVHD, PFS, or OS. The combined use of rituximab during the conditioning regimen may be regarded as an effective strategy for preventing EBV reactivation after rATG - based haplo - HSCT in the era of letermovir for CMV prophylaxis.

## Data Availability

The datasets used and/or analysed during the current study are available from the corresponding author on reasonable request.
